# Symptoms and Physical Exam Findings in Sexual Assault-related Non-fatal Strangulation

**DOI:** 10.5811/westjem.2021.2.50919

**Published:** 2022-02-08

**Authors:** Hannah H. Spungen, Karen M. Bryan, Carolyn J. Sachs, Malinda J. Wheeler

**Affiliations:** * UCLA Center for Health Sciences, Department of Emergency Medicine, Los Angeles, California; † UCLA David Geffen School of Medicine, Los Angeles, California; ‡ Forensic Nurse Specialists, Long Beach, California

## Abstract

**Objective:**

Our goal was to investigate the frequency of specific signs and symptoms following sexual assault-related non-fatal strangulation (NFS) and to explore the interaction between assault characteristics and physical exam findings.

**Methods:**

This retrospective observational study included all adults (>18 years) reporting strangulation during sexual assault who presented for a forensic sexual assault exam at one of six urban community hospitals contracted with a single forensic nurse agency. Demographic information, narrative elements, and physical exam findings were abstracted from standardized sexual assault reporting forms. We analyzed data with descriptive statistics and compared specific variables using chi-square testing.

**Results:**

Of the 580 subjects 99% were female, with a median age of 27 (interquartile range 22–35 years). The most common injury location was the neck (57.2%), followed by the mouth (29.1%). We found that 19.1% of the victims had no injuries evident on physical exam and 29.8% reported a loss of consciousness. Eye/eyelid and neck findings did not significantly differ between subjects who reported blows to the head in addition to strangulation and those who did not. The time that elapsed between assault and exam did not significantly correlate with the presence of most head and torso physical exam findings, except for nose injury (*P* = 0.02).

**Conclusion:**

Slightly more than half of the victims who reported strangulation during sexual assault had visible neck injuries. Other non-anogenital findings were present even less frequently, with a substantial portion of victims having no injuries documented on physical exam. The perpetrators’ use of blows to the head may account for many of the non-anogenital injuries observed, but not for the neck and eye/eyelid injuries, which may be more specific to non-fatal strangulation. More research is needed to definitively establish strangulation as the causal mechanism for these findings, and to determine whether any long-term neurologic or vascular sequelae resulted from the observed injuries.

## INTRODUCTION

While most emergency physicians (EP) rarely perform forensic sexual assault (SA) exams, the emergency department (ED) remains a critical access point for SA referral and resources, particularly in rural areas or in departments where a sexual assault nurse examiner (SANE) is not available on-call.^1^ Thus, it is imperative that EPs be familiar with the patterns of injury and morbidity associated with SA-related complaints, particularly those injuries that are associated with airway or circulatory compromise. Strangulation is one such potentially lethal mechanism of injury wherein external pressure applied to a victim’s neck ultimately obstructs the airway or cerebral blood flow.

Strangulation is not uncommonly reported in the course of SA. Zilkens et al (2016) estimated the frequency of non-fatal strangulation in SA victims to be about 7%,^2^ while McQuown et al (2016) reported an incidence of 12% among a similar population, noting that 97% of these cases had “significant risk for lethality.”^3^ Medical sequelae of strangulation have been well-documented and range from difficulty speaking and sore throat to laryngeal fracture, pulmonary edema, carotid dissection, stroke, coma, and death.^1^,^4^,^5^ Moreover, victims of intimate partner violence (IPV) who report a history of non-fatal strangulation have been shown to be at 7.48-fold greater risk of death by homicide than cohort-matched controls,^6^ making non-fatal strangulation an important prognostic indicator for recidivism and mortality.

Despite the consistency with which non-fatal strangulation is reported in SA survivors and the potential severity of risk that a history of strangulation confers, little is known about the specific injury patterns resulting from SA-related strangulation. Plattner et al (2003) attempted to generate a classification system for strangulation-related injury severity based on a retrospective chart review of 134 cases but went on to note that 95 (71%) of these cases did not fit criteria for just one category of classification.^7^ Another review of 300 domestic violence cases in San Diego County, California, noted that 50% of strangulation victims had no visible injury,^8^ a phenomenon that has historically led to trivialization of the violence that transpired (and potential medical sequelae thereof) by both law enforcement and medical personnel.^9^,^18^

Complicating matters further, medical documentation has been shown to significantly impact outcomes in the minority of SA cases that do go to trial,^10^ although there is a relative paucity of literature describing how often physical exam findings are documented in non-fatal strangulation during SA. In this study we sought to determine the frequency and characteristics of various symptoms and physical exam findings present in SA victims reporting strangulation, and to describe how these findings correlate with the mechanism of strangulation described in the victim’s account of the assault.

## METHODS

### Study Design

We performed a retrospective observational study describing the demographics, assault characteristics, and signs/symptoms present among adult SA victims presenting for forensic evaluation between January 2006– May 2019. An exemption to informed consent was granted by our institutional review board due to the absence of any personally identifying information recorded in our dataset.

### Study Setting and Population

The study population consisted of all adult (>18 years) SA victims reporting strangulation who were examined by a single forensic nurse examination agency contracted with six urban, community hospitals in the Southern California area between January 1, 2006–May 30, 2019. We excluded those victims who were unsure whether they had been strangled due to loss of consciousness, amnesia related to drug/alcohol use, or blunt head injury. We included those victims who reported strangulation even if a strangulation addendum form (as described in the study protocol) was not attached to their record. We excluded those victims whose recollection of the assault was so unclear, or so sparsely documented, that it was uncertain whether they had been strangled.

**Population Health Research Capsule**

What do we already know about this issue?

*Strangulation is a common injury sustained during sexual assault that can cause injury ranging from temporary pain or dyspnea to carotid dissection, coma, or death.*

What was the research question?

*What is the frequency, nature, and severity of the injuries associated with non-fatal strangulation in survivors of sexual assault?*

What was the major finding of the study?

*About 57% of subjects reporting strangulation during their assault had an associated positive finding on physical exam of the neck. Almost 20% had no positive physical exam findings.*

How does this improve population health?

*Understanding the injury patterns in sexual assault is essential to providing sensitive, trauma-informed care to this highly vulnerable population.*

### Study Protocol

The patient exams were performed by 38 SANE professionals who had been trained in accordance with the educational standards for the Office of Emergency Services in California and the International Association of Forensic Nurses. All exams were documented using the State of California Emergency Management Agency (Cal EMA) Form 2–923. The median number of exams performed by each nurse examiner was six, with an interquartile range of 2–12.75. During the forensic examination, all study subjects underwent a standardized interview, external physical exam with photo documentation, pelvic exam with photo documentation (unless specifically declined), and external anogenital exam with photo documentation (unless specifically declined). The Cal EMA 2–923 form contents were entered into a database management software TACT (Thorough Assault Case Tracking, Infosys Business Solutions, Bengaluru, India), which was then used to retrospectively identify all eligible subjects flagged as reporting strangulation during the assault. We then used the original Cal EMA 2–923 form as the source document for study data abstraction. Study variables were entered into a spreadsheet in Microsoft Excel (Microsoft Corporation, Redmond, WA).

### Study Variables

We collected demographic data including the victims’ gender, age, and race. The method of strangulation (eg, manual, ligature) the perpetrator’s relationship to the victim, and the victim’s pregnancy status were also reported. The presence of weapons (including type, if applicable), alcohol or drug use in the 24 hours preceding the assault, loss of consciousness, and a history of blows to the head during the assault were recorded, along with the presence or absence of specific symptoms: nausea/vomiting; breathing difficulty; urinary incontinence; difficulty swallowing; and voice changes. Each of these items was specifically included as a discrete question on the Cal EMA 2–923 form. The presence of any physical exam finding was reported for the following categories: face; head (excluding face); eyes/eyelids; ear; nose; mouth; under chin; neck; chest; and shoulders. We recorded the time that elapsed between the assault and the victim’s presentation as a quantitative variable. In cases where the assault occurred over several hours, the midpoint of the given time range between assault and exam was recorded. All time intervals were rounded to the nearest half hour.

### Data Analysis

Spreadsheet data were imported and analyzed with descriptive statistics using R version 3.6.3 (The R Foundation for Statistical Computing, Vienna, Austria). In addition to calculating percentage of positive physical exam findings in the entire study population, we performed several exploratory analyses to evaluate whether injury patterns differed based on specific assault characteristics. First, we calculated the difference in percentage of positive physical exam findings between victims who reported blows to the head vs those who did not, along with a 95% confidence interval (CI), for each physical exam category. Next, we calculated a difference in percentage between IPV vs a collapsed non-IPV cohort (including acquaintance, stranger, and “other” categories) along with a 95% CI for each physical exam category. Finally, a chi-square test with Yates’ continuity correction was performed to evaluate for interaction between selected study variables for hypothesis-generating purposes. For continuous variables, data was first logarithmically transformed to generate a more normal distribution and then broken up into tertiles. The tertiles were treated as discrete categories and subjected to chi-square testing as previously described.

## RESULTS

**Figure 1:**
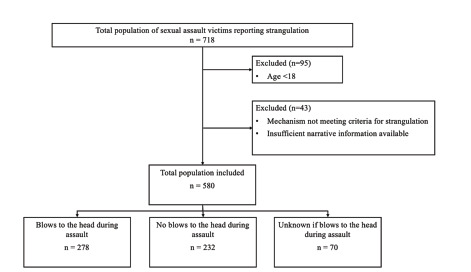
Flow diagram of study population inclusion showing breakdown of subjects who reported blows to the head, including a breakdown of the number of subjects who also reported blows to the head during the assault.

We identified 623 subjects **≥**18 years of age in the TACT database search, which returned all SA victims who were flagged as reporting strangulation. Ultimately, we included 580 of these subjects in the study database (see Figure 1 for exclusions). The demographics of included subjects are summarized in Table 1, as are the specific characteristics of the assault narrative.

The frequency of specific symptoms and of positive physical exam findings in specific anatomic locations is shown in Table 2. We found that 19.1% of subjects had no positive physical exam findings in any category. The difference in percentages of positive physical exam findings for victims who experienced blows to the head vs those who did not varied in magnitude, although it was always positive (ie, with higher percentages in the “blows to the head” subgroup). The data can be viewed in Supplemental Table e1. Notably, the percentage of positive findings was roughly similar (based on 95% CI) for the eyes/eyelids and neck physical exam categories. Injury patterns in IPV victims were similar to those in non-IPV victims for most physical exam categories. Exceptions included under chin (28.6% positive findings in IPV vs 15.6% positive findings in non-IPV), chest (29.7% positive findings in IPV vs 18.5% positive findings in non-IPV), and shoulders (28.6% positive findings in IPV vs 15.3% positive findings in non-IPV). This data can be viewed in Supplemental Table e2.

The interactions between selected assault characteristics and positive physical exam findings of interest for hypothesis-generating purposes are shown in Table 3. In addition, we evaluated the interaction between race and physical exam findings using Pearson’s chi-square test. The only physical exam variables that showed significant interaction with race included under chin, chest, and shoulders (all of which had *P* <0.05; complete results are available in Table e1). See Supplemental Table e3 for complete data.

## DISCUSSION

Neck findings (ecchymoses, erythema, swelling, abrasions, lacerations, or petechiae) were present in 57.2% of our study population, which is higher than previous estimates of physical exam findings in non-fatal strangulation^2^,^8^,^11^; however, most of the available literature is not specific to a SA population, and SA perpetrators may be more likely to use violent and/or prolonged strangulation as a means of subjugation. Another possibility is that our forensic examiners detected higher injury rates due to the thorough nature of their training and systematic documentation requirements. The fact that almost 43% of victims reporting non-fatal strangulation had no visible neck exam findings on a rigorous and systematic examination nevertheless reinforces existing literature demonstrating the inconsistency with which this mechanism of injury is associated with physical exam findings, even by the most highly trained of forensic nurse examiners. Apart from neck injury, the remainder of exam findings (excluding anogenital exam, which was not included in our dataset) were present in less than 30% of victims for each category, suggesting that assault-related strangulation in this population is not necessarily associated with a high frequency of visible comorbid head/trunk injuries.

We calculated the difference in percentage between physical exam findings in victims who reported blows to the head vs those who did not to ascertain the extent to which our findings might be accounted for by physical blows rather than strangulation. Because our study was not designed to isolate the exam findings among strangled vs non-strangled SA victims, we used this comparison instead as an indicator of whether our results might be systematically biased by the presence of injuries from an alternative mechanism. Unsurprisingly, we found that subjects who reported battering had higher rates of positive findings in most categories. More interesting were those categories that did *not* show a significant percent difference among the battering vs non-battering populations, namely, for eyes/eyelids and neck. The similarity across these two populations suggests that neck findings and eye/eyelid findings might be specific to strangulation, although further research involving a control population would be required to definitively demonstrate this.

Another variable that might be expected to have an impact upon injury patterns is the use of ligature vs manual strangulation. Few comparisons or conclusions can be drawn about the impact of ligature vs manual strangulation on injury patterns from our data, as only a small number of assailants (2.6%) used a ligature. This low prevalence of ligature use has been observed in other populations of SA-related non-fatal strangulation, as well.^2^ Other factors that had significant associations with loss of consciousness, which has widely been used as an indicator of strangulation severity,^3^,^6^,^12^ included positive physical exam findings for most exam location categories, the type of perpetrator, the victim expecting to die, and the perpetrator having a weapon. While none of these associations are not surprising, it is notable that one of the most robust correlations we found was between loss of consciousness and eye/eyelid findings (*P* <0.001). Petechiae and subconjunctival hemorrhage (SCH) have both been reported as potential candidates for predictors of strangulation severity.^2^,^7^,^12^ While the frequency of reported eye/eyelid findings in our population was only 12.8%, the significant association between eye/eyelid findings and loss of consciousness supports the previously proposed hypothesis that there may be a threshold for cerebral venous outflow obstruction after which petechiae are more likely to appear, and that this threshold may exceed that amount of time necessary to produce loss of consciousness;^7^ however, because eye/eyelid findings in our dataset were not limited to SCH/petechiae, more granular comparison of different types of findings (SCH, petechiae, periorbital ecchymosis, conjunctival injection, etc) would be needed to determine whether this association holds true for more specific findings. Likewise, much more data would be needed in the form of prospective comparisons of strangulation vs non-strangulation patients to determine whether any of the aforementioned mechanisms are causative as opposed to just correlative.

Our sample demonstrates a high prevalence of IPV-related assaults, which is consistent with prior epidemiologic studies of assailant types in non-fatal strangulation,^6^ although surprisingly, acquaintances represented the most frequent assailant type in our sample. Because the perpetrator relationship is self-reported by the victim, more complex interpersonal relationships between victim and perpetrator may have escaped either the coding by the forensic nurse examiner or the categorization scheme that we used to report the perpetrator category. Additional data would be required to determine whether this phenomenon holds true in the more general population of SA victims.

Much of the literature related to non-fatal strangulation has grown out of the pioneering work by Strack et al (2001) in their landmark, 300-subject case series of IPV-related, non-fatal strangulation, which showed even higher rates of reported strangulation without physical exam findings, on the order of 50% with no visible injury.^8^ While only 19.1% of the subjects in our study had no physical exam findings whatsoever, there are several methodological differences that may account for this discrepancy. First, our study data was gathered by forensically trained nurse specialists as opposed to police officers, the former of whom are much more highly trained and adept at recognizing physical injury patterns in assault. Secondly, we had a substantial percentage of subjects missing one or more physical exam categories, which may have introduced substantial bias. Lastly, our population consisted of those victims who sought medical assessment as opposed to those victims whose cases were prosecuted. It is possible that victims who sought forensic assessment in a healthcare setting had a greater symptom burden and/or more severe injuries than those retrospectively analyzed from prosecution cases. It is also important to note that our population was not restricted to IPV, which may render our results more externally generalizable than the Strack case series.

We also used our data to explore a hypothesized differential prevalence of physical exam findings in people of color. Previous studies have shown that Black women experience higher rates of SA than their White counterparts within specific age and socioeconomic subcategories.^13^,^14^ Moreover, it has also been demonstrated that Black and Brown victims of SA have decreased rates of visible injury than their White counterparts.^14^,^15^ Our data corroborated these prior observations for specific categories, namely that race was significantly associated with the presence or absence of neck, under chin, and chest findings. Although the chi-square test we performed was limiting in that it did not indicate which populations were more or less likely to have positive findings, it does suggest that this is a topic worth examining on a more granular level.

## LIMITATIONS

Our study had several significant limitations in addition to those already addressed. First and foremost, we only studied individuals over the age of 18; thus, our data does not necessarily generalize to a child/teenage population. Additionally, there is substantial inherent sampling bias in any study of individuals voluntarily presenting for a forensic SA. Estimates of the proportion of SA victims who present for forensic exam range from around 14–21%^13^,^16^,^17^; thus, our observations are not necessarily generalizable to the broader population of SA survivors. This bias could have affected our conclusions in divergent ways. Our data may reflect a greater frequency of injuries if victims who sought care did so due to more substantial injury/symptom burden than the general population; however, our study population also excluded those individuals too acutely ill to consent to a forensic examination, which would shift our conclusions toward underestimating injury burden. Although the latter category likely represents a small percentage of total SA-related non-fatal strangulation victims, more research is needed to determine the characteristics and prevalence of injury patterns in this more critically ill cohort.

Finally, between 9–12% of subjects were missing data for each physical exam category. The similarity across all exam categories likely reflects the portion of exam reports wherein a physical exam section was erroneously omitted or left blank. Although this likely introduced a degree of bias into our results (as described above), we felt it was more important to include the subjects with missing exam data to gain a more accurate picture of assault characteristics and victim demographics.

## CONCLUSION

In sexual assault survivors who have been strangled, the presence of non-anogenital physical exam findings is inconsistent, and the absence thereof does not undermine the veracity of a reported history of strangulation. Neck findings were seen much more commonly than any other facial, head, or torso injuries. The time elapsed between exam and assault did not significantly interact with most of the exam finding categories, nor did the victim’s age. Further research is necessary to determine a causal relationship between the variables we examined and specific physical exam findings.

## Supplementary Information



## Figures and Tables

**Table 1:** Demographics and assault characteristics of sexual assault victims who experienced non-fatal strangulation.

	Frequency (N = 580)	%
Demographics		
Gender		
Female	574	99
Male	6	1
Age (years)*		
18–30	356	61.3
31–40	136	23.4
41–50	57	9.8
51–60	24	4.1
>= 61	7	1.2
Race		
Asian	33	5.7
Black	125	21.7
Hispanic	203	35.2
Other	13	2.3
Unknown	4	0.7
White	202	35.1
Characteristics of Assault		
Assault to Presentation Time (hours)*	10 (5–22)	
Perpetrator		
Acquaintance	241	41.6
Stranger	91	15.7
IPV	192	33.1
Unknown	48	8.3
Other	8	1.4
Method		
Ligature	15	2.6
Manual	506	87.2
Other	8	1.4
Not documented	51	8.8
Alcohol use (up to 24 hours prior to assault)		
Yes	242	41.7
No	347	58.1
Unknown	1	0.2
Drug use (up to 24 hours prior to assault)		
Yes	188	32.4
No	381	65.7
Unknown	11	1.9
Pregnant		
Yes	33	5.7
No	529	91.2
Unknown	18	3.1
Hair pulled		
Yes	128	22.1
No	345	59.5
Unknown	107	18.4
Blows to head		
Yes	278	47.9
No	232	40.0
Unknown	70	12.1
Perpetrator threatened to kill victim		
Yes	168	29.0
No	321	55.3
Unknown	91	51.7
Victim expected to die		
Yes	216	37.2
No	240	41.4
Unknown	124	21.4
Weapon present		
Gun	57	9.8
Gun and knife	10	1.7
Knife	77	13.3
None	377	65
Other	47	8.1
Unknown	12	2.1

* Expressed as median (interquartile range).

*IPV*, intimate partner violence.

**Table 2:** Symptoms and physical exam findings present in non-fatally strangled sexual assault victims.*

	Yes	No	Unknown/Missing
Symptoms reported			
Loss of consciousness	173 (29.8%)	402 (69.3%)	5 (0.9%)
Breathing difficulty	253 (43.6%)	240 (41.4%)	87 (15.0%)
Voice changes	212 (36.6%)	280 (48.3%)	88 (15.2%)
Swallowing difficulty	211 (36.4%)	280 (48.3%)	89 (15.3%)
Nausea and/or vomiting	122 (21.0%)	361 (62.2%)	97 (16.7%)
Urinary incontinence	41 (7.1%)	450 (77.6%)	89 (15.3%)
Physical exam findings present			
Face	141 (24.3%)	366 (63.1%)	73 (12.6%)
Eyes/eyelids	74 (12.6%)	455 (78.4%)	51 (8.8%)
Nose	45 (7.8%)	476 (82.1%)	59 (10.2%)
Ears	47 (8.1%)	473 (81.6%)	60 (10.3%)
Mouth	169 (29.1%)	355 (61.2%)	56 (9.7%)
Head	67 (11.6%)	458 (79.0%)	55 (9.5%)
Neck	332 (57.2%)	177 (30.5%)	71 (12.2%)
Under chin	117 (20.2%)	407 (70.2%)	56 (9.7%)
Chest	132 (22.8%)	393 (67.8%)	55 (9.5%)
Shoulders	116 (20%)	409 (70.5%)	55 (9.5%)

* Expressed as number of subjects (%)

**Table 3:** Interaction between selected assault characteristics and physical exam findings in sexual assault-related non-fatal strangulation.≠

	LOC p-value	Log(age) tertile p-value	Log (assault-exam interval) tertile p-value
Physical exam category			
Face	0.06869	0.2192	0.5242
Eyes/eyelids	0.001417***	0.4731	0.87
Nose	0.0001602***	0.9806	0.02281*
Ear	0.00002694***	0.2376	0.2367
Mouth	0.4535	0.8524	0.5563
Under chin	0.01538*	0.5242	0.4369
Chest	0.3602	0.6204	0.6957
Shoulders	0.4568	0.01223*	0.09011
Neck	0.007889**	0.5244	0.9678
Head	0.8797	0.358	0.06237
Assault characteristics			
Method	0.3866	0.09953	0.6646
Perpetrator	0.0009043***	0.003762**	0.0004507***
Presence of weapon	0.04543*	0.2369	0.1565
Victim expected to die	0.01923*	0.01124	0.3067
Perpetrator threatened to kill victim	0.327	0.07986	0.501

≠ Using Pearson’s chi-squared test with Yates’ continuity correction.

* *P* <0.05.

** *P* <0.01.

*** *P* <0.005.

*LOC*, location.
