# Supplementary material for: Symptoms and Physical Exam Findings in Sexual Assault-related Non-fatal Strangulation
Source: West J Emerg Med. 2022 Feb 8;23(2):268–75. doi: 10.5811/westjem.2021.2.50919 (PMC8967470; doi:10.5811/westjem.2021.2.50919)
Supplement: Supplementary file 1 [file wjem-23-268-s001.docx]

**Supplemental Table e1: Positive physical exam findings subdivided by presence or absence of blows to the head**

| **Location of**  **exam findings** | **% subjects with positive findings** | | | | **Difference in percentage, blows to head vs. no blows to head (95% CI)** | |
| --- | --- | --- | --- | --- | --- | --- |
|  | **Overall** | **History of blows to head** | **No history of blows to head** | **Unknown if blows to head** |  |  |
| Face | 24.3 | 36.0 | 14.7 | 12.6 | 21.3 (13.9, 28.5)* |  |
| Eyes/Eyelids | 12.8 | 15.1 | 12.1 | 8.8 | 3.0 (-3.1, 9.0) |  |
| Nose | 7.8 | 10.4 | 5.6 | 10.2 | 4.8 (0.03, 9.6)* |  |
| Ears | 8.1 | 11.9 | 5.2 | 10.3 | 6.7 (1.9, 11.6)* |  |
| Mouth | 29.1 | 39.9 | 22.4 | 9.7 | 17.5 (9.5, 25.2)* |  |
| Under Chin | 20.2 | 27.0 | 15.9 | 9.7 | 11.0 (3.9, 18.0)* |  |
| Chest | 22.8 | 28.8 | 19.0 | 9.5 | 9.8 (2.4, 17.1)* |  |
| Shoulders | 20.0 | 26.3 | 16.4 | 9.5 | 9.9 (2.7, 16.9)* |  |
| Neck | 57.2 | 63.7 | 61.6 | 12.2 | 2.0 (-6.4, 10.5) |  |
| Head | 11.6 | 18.7 | 5.6 | 9.5 | 13.1 (7.6, 18.7)* |  |

*95% CI does not include zero

**Supplemental Table e2: Percentage of positive physical exam findings in IPV versus non-IPV victims in sexual assault-related non-fatal strangulation**

| **Physical Exam Category** | **% positive physical exam findings** | | | **Difference in percentage (95% CI)** |
| --- | --- | --- | --- | --- |
|  | **Total** | **IPV victims** | **Non-IPV victims** |  |
| Face | 24.3 | 31.3 | 19.4 | 11.9 (4.3, 19.8)* |
| Eyes/eyelids | 12.8 | 14.0 | 14.7 | -0.8 (-7.0, 6.0) |
| Nose | 7.8 | 7.4 | 9.4 | -2.0 (-6.9, 3.6) |
| Ear | 8.1 | 7.8 | 8.1 | -0.3 (-4.8, 5.0) |
| Mouth | 29.1 | 31.3 | 28.0 | 3.2 (-4.7, 11.4) |
| Under chin | 20.2 | 28.6 | 15.6 | 13.0 (5.8, 20.7)* |
| Chest | 22.8 | 29.7 | 18.5 | 11.2 (3.7, 19.0)* |
| Shoulders | 20.0 | 28.6 | 15.3 | 13.3 (6.1, 21.0)* |
| Neck | 57.2 | 62.5 | 53.8 | 8.7 (-0.0006, 17.2) |
| Head | 11.6 | 14.1 | 10.7 | 3.4 (-2.3, 9.7) |

*95% CI does not cross zero

**Supplemental Table e3: Interaction between race and physical exam findings in sexual assault-related non-fatal strangulation^≠^**

| **Physical exam category** | **p-value** |
| --- | --- |
| Face | 0.4778 |
| Eyes/eyelids | 0.1934 |
| Nose | 0.1832 |
| Ear | 0.08073 |
| Mouth | 0.6637 |
| Under chin | 0.005536** |
| Chest | 0.01552* |
| Shoulders | 0.1975 |
| Neck | 0.0441* |
| Head | 0.3804 |

^≠^Using Pearson’s Chi-squared test

*p<0.05

**p<0.01
